# Signs and symptoms of serious illness in adults with acute abdominal pain presenting to ambulatory care: a systematic review

**DOI:** 10.3399/BJGPO.2023.0245

**Published:** 2024-09-04

**Authors:** Anouk Tans, Thomas Struyf, Rune Geboers, Toon Smeets, Yorick Asselbergh, Emmanuel Declerck, Luca Bloemen, Ann van den Bruel

**Affiliations:** 1 Department of Public Health and Primary Care, KU Leuven, Leuven, Belgium; 2 Biomedical Sciences, Faculty of Medicine, KU Leuven, Leuven, Belgium; 3 Interuniversity Partnership for GP Training, KU Leuven, Leuven, Belgium

**Keywords:** diagnosis, systematic review, gastroenterology, abdominal pain, ambulatory care

## Abstract

**Background:**

Acute abdominal pain is a common complaint, caused by a variety of conditions, ranging from acutely life-threatening to benign and self-limiting, with symptom overlap complicating diagnosis. Signs and symptoms may be valuable when assessing a patient to guide clinical work.

**Aim:**

Summarising evidence on the accuracy of signs and symptoms for diagnosing serious illness in adults with acute abdominal pain in an ambulatory care setting.

**Design & setting:**

We performed a systematic review, searching for prospective diagnostic accuracy studies that included adults presenting with acute abdominal pain to an ambulatory care setting.

**Method:**

Six databases and guideline registers were searched, using a comprehensive search strategy. We assessed the risk of bias, and calculated descriptive statistics and measures of diagnostic accuracy. Results were pooled when at least four studies were available.

**Results:**

Out of 18 923 unique studies, 16 studies with moderate to high-risk bias were included. Fourteen clinical features met our criteria, including systolic blood pressure <100 mmHg (positive likelihood ratio [LR+]7.01), shock index >0.85, uterine cervical motion tenderness (LR+5.62 and negative likelihood ratio [LR-]8.60), and a self-assessment questionnaire score >70 (LR+12.20) or <25 (LR-0.19). Clinical diagnosis made by the clinician had the best rule-in ability (LR+24.6).

**Conclusions:**

We identified 14 signs and symptoms that can influence the likelihood of a serious illness, including pain characteristics, systemic signs, gynaecological signs, and clinician’s overall assessment. The risk of bias was moderate to high, leading to uncertainty and preventing us from making firm conclusions. This highlights the need for better research in this setting.

## How this fits in

The diagnostic accuracy of signs and symptoms used in an ambulatory care setting is mainly based on research of hospitalised patients. Unfortunately, these patient populations are vastly different from those presenting to ambulatory care settings. In addition, in clinical practice, our decision-making process is based on history taking and clinical investigation, which is traditionally taught in medical school. Assessing these signs and symptoms critically and evaluating their diagnostic accuracy in an ambulatory specific setting is important to maximise patient safety in ambulatory care and minimise unnecessary investigations and referrals.

## Introduction

Abdominal pain affects nearly every person at least once in their lifetime.^1^ Annually, 56 per 1000 patients present with acute abdominal pain to GPs,^2^ and abdominal symptoms are recorded in 10.1% of consultations in general practice.^3^ It is one of the most common reasons for referral to secondary care,^4^ accounting for up to 10% of all emergency department (ED) visits.^5–7^

Abdominal pain can be caused by a variety of conditions, ranging from acutely life-threatening to benign and self-limiting.^8^ Most common causes are acute gastroenteritis, appendicitis, choledocholithiasis, acute pancreatitis, and neoplasms.^1,9^ In young females, gynaecological conditions such as ectopic pregnancy and pelvic inflammatory disease should also be considered.^10,11^ Overall 1-year mortality rates in patients with acute abdominal pain are approximately 3% in general practice.^8^ Mortality rates are higher in patients presenting to the ED; 2% at 24 hours and 4% at 7 days.^12^ Timely recognition of serious illnesses causing acute abdominal pain is therefore important to limit morbidity and mortality.^13,14^

Clinicians in an ambulatory care setting, which is defined as any clinical setting where the patient is not admitted to a hospital, including general practice, walk-in clinics, or ED, strongly rely on signs and symptoms to guide their decision making and further work-up of a patient with acute abdominal pain.^1^ In addition, ambulatory care clinicians have to balance the risk of rapidly evolving causes with the risk of hospital admission and unnecessary treatments and referrals. Clinicians face substantial diagnostic uncertainty in patients presenting with abdominal pain in this setting.^8^ The diagnostic process should be strengthened by focusing on those signs and symptoms that are most accurate in either including or excluding serious conditions.

With this review, we aimed to identify and summarise available evidence on the accuracy of signs and symptoms to distinguish serious from non-serious causes of acute abdominal pain.

## Method

The protocol was registered a priori in PROSPERO (ID: 421510). We reported this study in accordance with the Preferred Reporting Items for Systematic Reviews and Meta-Analysis (PRISMA) guidelines.^15^

This article is part of a larger review, investigating all diagnostic tests for patients with acute abdominal pain presenting to an ambulatory care setting, including clinical features, blood tests, and biomarkers and imaging tests. In this article, we focused on signs and symptoms.

### Search strategy

The MEDLINE, Embase, DARE, CINAHL, Web of Science, and Cochrane Library databases were searched. The first search was undertaken from inception to December 2021. The search was updated in August 2023. The search strategy included MeSH/Emtree terms and free text. Additionally, we checked references of included studies and related reviews, as well as relevant guidelines (see Supplementary Appendix S1).

All articles were screened by two independent reviewers, first on title and abstract, followed by full text using Covidence online software (Covidence systematic review software). One reviewer screened all articles to ensure consistency, the second independent screening was done by a second reviewer. Conflicts were resolved by a third independent reviewer. Selection criteria were defined a priori and all reviewers were trained on a subset of 20 articles.

### Inclusion criteria

We included cross-sectional prospective diagnostic accuracy studies on signs and symptoms in patients aged ≥18 years presenting with acute abdominal pain to an ambulatory care setting.

Ambulatory care settings included general practice, out-of-hours services, walk-in clinics, interface clinics, EDs, and other settings where patients were not admitted in a hospital or another facility.

Acute abdominal pain was defined as pain (of any intensity) anywhere between the chest and groin, present for a maximum of 14 days. Studies including participants of all ages were eligible when at least 50% of the population was aged ≥18 years or when they reported results for adults separately.

### Exclusion criteria

Given the low incidence of serious causes of acute abdominal pain, we excluded studies with a sample size of <50 participants. Such studies would include only a few subjects with the target condition and would be prone to selection bias.^16,17^

Studies including patients with known active abdominal malignancies or chronic pre-existing gastrointestinal diseases (for example, inflammatory bowel syndromes) were excluded.

Case reports, case series, retrospective studies, conference abstracts, systematic reviews, and case-control studies were also excluded. Whereas nested case-control studies were eligible.

No language restrictions were applied a priori. During article selection, studies were excluded when no trustworthy translation to English or Dutch was available.

### Quality assessment

QUADAS-2 was used to assess the risk of bias using RevMan (version 5.3). Quality assessment was performed by a first reviewer and checked by a second reviewer. Disagreements were resolved by a third independent reviewer through a consensus meeting.

### Data extraction and analysis

Data were extracted by all reviewers and checked by one second reviewer. Discrepancies were discussed and corrected. We reconstructed two-by-two tables based on the reported data. We calculated measures of diagnostic accuracy and their estimates of precision using RevMan.

Results were pooled using a bivariate model when at least four studies with acceptable clinical heterogeneity were available.

We calculated sensitivity, specificity, likelihood ratios (LRs), and their 95% confidence intervals (CIs) for each index test (that is, a sign or a symptom). We considered tests to be useful in lowering the likelihood of a target condition in ambulatory care when the negative LR was <0.20. Diagnostic tests were considered useful as ‘red flags’ when their positive LR was ≥5.0.^18,19^

## Results

**Figure 1.**
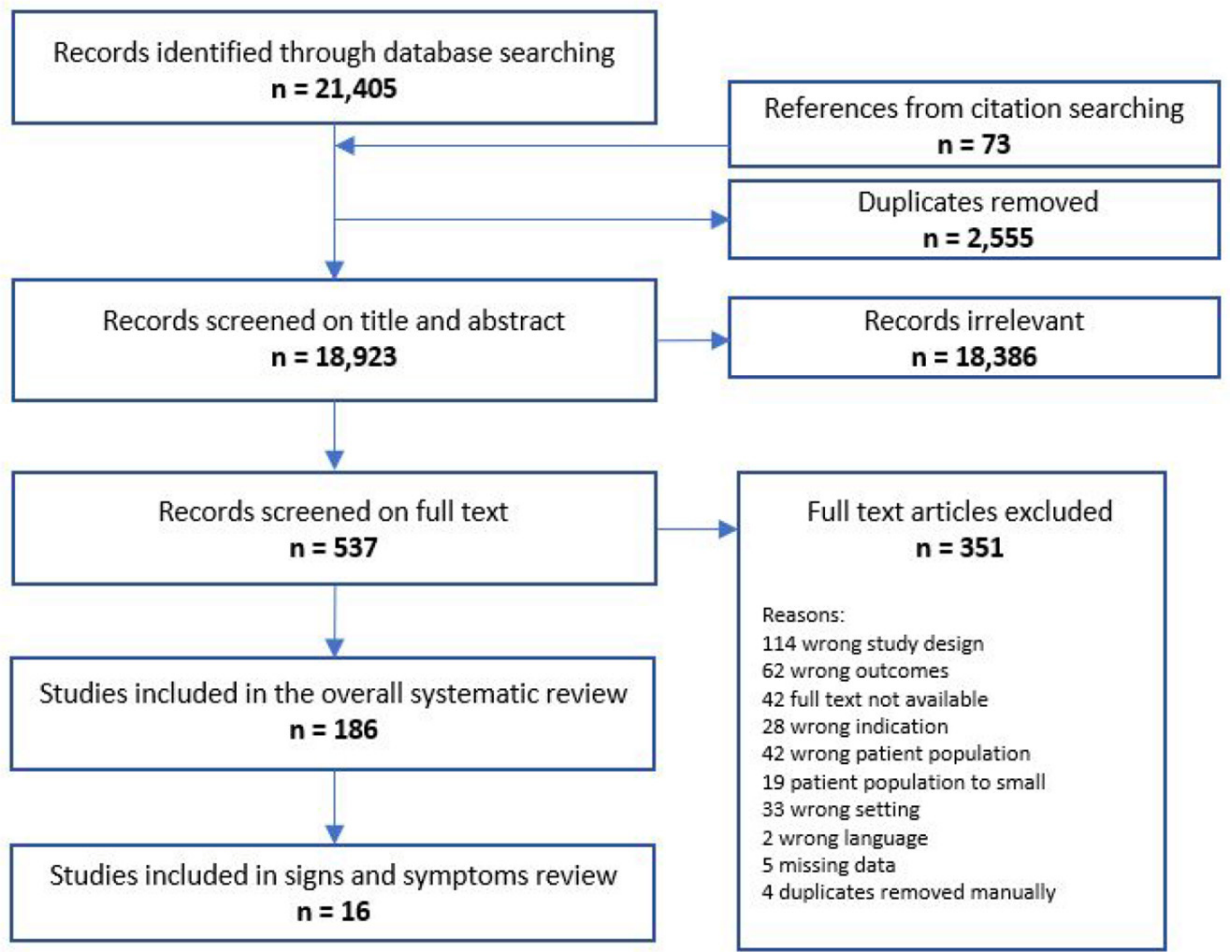
Flow diagram for selection of studies

We identified 18 923 unique studies, of which 537 were selected for full-text screening. We included 186 studies in the overall review. Sixteen studies reported data on signs and symptoms (Figure 1). Due to no available trustworthy translations, two studies were excluded. Here, we report the findings of these 16 studies.^20–35^

### Study population

All studies were conducted at the ED, none in general practice. The total number of patients was 6004 ranging from 86^20^ to 1154^21^ per study. Participants’ mean age across studies varied from 19.5^20^ to 52.3^22^ years. Eighty-five per cent of all participants were female, ranging from 22%^23^ to 100%.^24–28^

Target conditions were appendicitis,^20,21,23,29–33^ ectopic pregnancy,^25–28^ diverticulitis,^21^ admittance to hospital,^22,34^ surgical treatment,^22,34^ acute cholecystitis,^35^ and pelvic inflammatory disease.^24^

The incidence of target conditions differed substantially between studies, for example, ranging from 8.2%^20^ to 85.0%^23^ for appendicitis. This was mainly caused by differences in inclusion and exclusion criteria, with some studies selecting patients with a high suspicion of appendicitis (for example, appendicitis as a top three differential diagnosis in the work-up of the patient), while other studies applied broader inclusion criteria (for example, all patients presenting with any acute abdominal pain). Study characteristics are displayed in Table 1.

**Table 1.** Characteristics of included studies

Study reference	Setting	Population, *n*; mean age, years; female, %; incidence, %	Inclusion criteria	Exclusion criteria	Index tests	Reference standard
**Appendicitis**						
^23^	ED; N/A	100; 34; 22; 85	Aged >15 years; abdominal pain suggestive of acute appendicitis	Abdominal pain elsewhere	Speed bump sign	Pathology
^20^	ED; US	86; 19.5; 61.6; 26.7	Chief complaint of RLQ pain	Pregnancy; previous abdominal surgery; immunocompromised	Abdominal thermography RLQ versus LLQ (difference in temperature, °c)	Pathology; follow-up data after 2–8 weeks
^33^	Surgery ED; Spain	192; 21.8; 53; 52.6	RLQ pain <7 days	Ultrasound could not be performed; clear diagnosis of appendicitis	Rebound tenderness, guarding	Radiological score system for appendicitis; leucocytosis >10 500 mm^3^ + left shift >75% neutrophils
^32^	ED; the Netherlands	942; 37; 55; 30.1	Aged >18 years; non-traumatic abdominal pain >2 hours <5 days	Discharged without imaging; haemorrhagic shock; pregnancy	History of RLQ pain; pain migration to RLQ; pain on movement; anorexia; nausea; progressive pain; history of fever; vomiting; diarrhoea; tenderness RLQ; tenderness RLQ only; rebound tenderness; rigidity; abdominal tenderness; rectal tenderness	Expert panel based on imaging reports, clinical presentation, pathology, and follow-up data
^29^	ED; US	261; 35; 67.8; 20.3	Non-traumatic abdominal pain <72 hours; acute appendicitis included in top three most likely diagnoses	Pregnancy; abdominal trauma; abdominal surgery within 7 days prior to inclusion; non-English speaking	Clinical diagnosis	Imaging report; pathology
^30^	Surgery ED; Germany	400; 22; 63; 23	Aged >6 years; <7 days acute abdominal pain	Recent abdominal trauma <4 weeks; recent abdominal surgery <4 weeks; appendectomy	Temperature >37.5°C	Pathology; medical chart data
^31^	ED; Denmark	226; 27; 62.4; 40.3	Aged ≥15 years; suspected appendicitis; <7 days abdominal pain	Known cancer; pregnancy; IBD; hepatitis; blood results known before physical examination; full anamnesis could not be obtained; radiological examination before blood samples where available	Clinical diagnosis	Surgical findings; pathology; imaging reports; follow-up data
^21^	ED; US	1154; 42; 58; appendicitis 8.2, diverticulitis 11.5, obstruction 4.20	Abdominal pain; CT of the abdomen was requested as part of their work-up	Not specified	Clinical diagnosis; RLQ pain; RLQ tenderness; LLQ pain; LLQ tenderness	CT
**Acute cholecystitis**						
^35^	ED; US	192; 38; 80.7; 60.4	RUQ or epigastric abdominal pain who underwent laparoscopic cholecystectomy	Not specified	Persistent abdominal pain; abdominal tenderness	Pathology
**Ectopic pregnancy**						
^27^	ED; US	378; 27.8–32.5; 100; 16.1	Abdominal pain and/or vaginal bleeding; positive urine B-HCG+ estimated gestational age <2 weeks	Evaluation for a complaint other than abdominal pain or vaginal bleeding	Shock index (heart rate/systolic blood pressure) >0.7; shock index >0.85; heart rate >100 beats per minute; systolic blood pressure <100 mmHg	Pathology; medical chart data; follow-up data
^25^	ED; US	486; 39; 100; 7.4	Abdominal pain or vaginal bleeding in first trimester of pregnancy	Haemodynamic instability; prior documentation of IUP on pelvic ultrasound; enrolled in a previous ED registry; gestational age ≥13 weeks	Foetal heart tones; tissue at cervical os; pain other than midline cramping; any abdominal pain; tissue passed by history; open cervical os; estimated gestational age <70 days; any abdominal tenderness; any pelvic abnormality; any adnexal tenderness; any vaginal bleeding; any cervical motion tenderness; any ectopic risk factors; abdominal peritoneal signs; definite cervical motion tenderness; discrete adnexal mass	According to criteria Table 1; follow-up data
^26^	ED; US	429; 25.3; 100; 7.2	Abdominal pain or vaginal bleeding in first trimester of pregnancy	Haemodynamic instable; prior documentation of IUP on pelvic ultrasound; enrolled in a previous ED registry; gestational age ≥13 weeks	High risk rule: any signs of peritoneal irritation/definite cervical motion tenderness; intermediate risk rule: no foetal heart tones, no tissue visible at the cervical os, and abdominal pain/tenderness	According to criteria Table 1; follow-up data
^28^	Gynaecological ED; France	262; not specified; 100; 59.5	Acute pelvic and/or vaginal bleeding in first trimester of pregnancy	History of chronic pelvic pain; non-French speaking; psychiatric or neurologic diseases	SAQ EP <25; SAQ >70; no diffuse abdominal pain; anal pain; no lumbar pain; unilateral pelvic pain; never experienced such pain before; no pain such as uterine contraction; pain provoked by palpation; pain on movement; pain provoked by coughing; sudden onset of pain; no fatigue; vomiting absent or unique; duration of bleeding >24 hours; bleeding absent or less than periods; no need for frequent change of sanitary towels; no evacuation of membranes; no pain during; evacuation of clots; no evacuation of clots; brown discharge	Laparoscopy; non-surgical algorithm based on transvaginal ultrasound and B-HCG measurement
**Pelvic inflammatory disease**						
^24^	Gynaecological ED; France	499; 32; 100; 14.6	Aged ≥18 years; presenting with acute pelvic pain	History of chronic pelvic pain; evolving intrauterine pregnancy of 15 weeks gestation or older; no knowledge of French; neurological or psychiatric disease impacting cognitive function; haemodynamic instability	Imprecise location of pain; diffuse pain; bilateral pelvic pain; left side pain; lateralised pain; pain in the uterus; pain radiating to thighs; pain radiating to ribs; pain radiating to stomach; intense pain; progressive pain; duration of pain >24 hours; ongoing pain; pain crises >30 minutes; pain provoked by coughing; pain provoked by palpation; awakened by pain; abnormal vaginal discharge; fatigue; constipation; no vaginal bleeding; scattered pain radiating and/or diffuse pain; insidious pain; peritoneal irritation; first model (four variables: scattered pain radiation and/or diffuse pain, insidious pain, peritoneal irritation, and abnormal vaginal discharge); second model (four variables: abnormal vaginal discharge, bilateral pelvic pain, constipation, and intrauterine device)	Laparoscopic/laparotomic diagnosis; transvaginal ultrasound or CT with typical images of PID; cultures of endocervical swabs being positive; based on non-invasive prediction rule of the CDC
**Surgical treatment and admittance to hospital**						
^22^	ED; Turkey	77; 52.3; 22; 85	Aged 16–65 years; acute non-traumatic abdominal pain <1 week	Resuscitation; history of diabetes; renal insufficiency; pregnancy; previous abdominal operation; analgesia; uncooperative	Pain with expiration; pain with inspiration; pain with coughing; pain with heel drop jarring	Clinical records of admission to hospital or surgery
^34^	ED; US	192; 47.7; 49.0; 76.6	Acute abdominal pain <48 hours that warrant CT	Recent surgery; pain of traumatic origin	Carnett’s sign; closed eye sign	CT; pathology; follow-up data

B-HCG = beta-human chorionic gonadotropin. CDC = Centers for Disease Control and Prevention. CT = computed tomography. ED = emergency department. EP = ectopic pregnancy. IBD = inflammatory bowel disease. IUP = intra-uterine pregnancy. LLQ = left lower quadrant. N/A = not available. PID = pelvic inflammatory disease. RLQ = right lower quadrant. RUQ = right upper quadrant. SAQ = self-assessed questionnaire.

### Risk of bias

**Figure 2.**
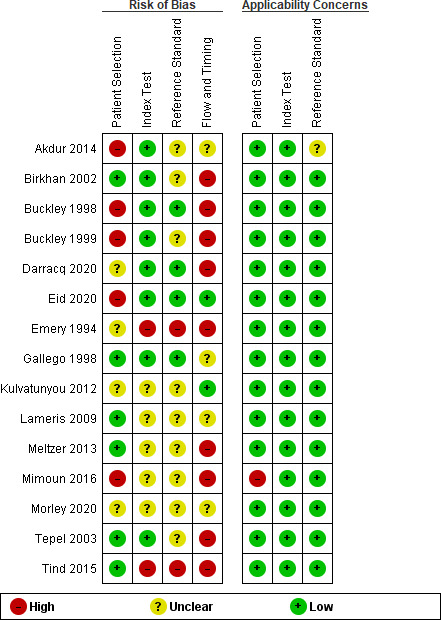
Risk of bias summary: QUADAS-2 risk of bias and applicability

An overview of the risk of bias assessment is presented in Figure 2. The overall risk of bias was moderate to high. A high risk of bias for patient selection was found in six studies due to strict exclusion criteria, for example, excluding patients with an extensive previous medical history.^22–26,28^

In 75% of studies there was uncertainty about blinding of the index test^21,28,29,32,35^ and reference standard.^21,22,27–30,32,35^ Three studies interpreted the results of the index test with the knowledge of the reference standard and vice versa.^20,21,31^

Concerning the reference standard, not all patients received the same, causing a high risk of bias in 62% of studies.^20,24–31,34^ There was uncertainty about the timing between the index test and reference standard due to bad reporting of the data, causing an unclear risk of bias in 37% of studies for flow and timing.^21,22,32,33^

Concerns about applicability was high in one study^28^ because patients were recruited and data were collected at the ED, but only data from patients who were later admitted to hospital were used.

### Diagnostic test accuracies

Out of the 16 studies included, 79 different signs and symptoms were identified as index tests. Index tests, for which at least one study reported LRs that match our prespecified criteria, are discussed in detail (see Supplementary Appendix S2).

#### Pain characteristics

Non-specified abdominal pain and lateralised/flank pain have a negative LR (LR-) of 0.17 (95% CI = 0.02 to 1.18)^25^ and 0.12 (95% CI = 0.02 to 0.84),^25^ respectively, for diagnosing ectopic pregnancy. Insidious pain and scattered/radiating pain had an LR- of 0.07 (95% CI = 0.03 to 0.18)^24^ and 0.19 (95% CI = 0.11 to 0.36),^24^ respectively, for diagnosing pelvic inflammatory disease (see Supplementary Appendix S2).

#### Systemic signs

Systolic blood pressure (BP) <100 mmHg and shock index (which is defined as heart rate/systolic BP) >0.85 are both red flags for appendicitis, with a positive LR (LR+) of 7.01 (95% CI = 2.39 to 20.58)^27^ and 13.15 (95% CI = 5.91 to 29.29),^27^ respectively (see Supplementary Appendix S2).

#### Gynaecological signs and symptoms

Cervical motion tenderness was a red flag for ectopic pregnancy in two studies, with an LR+ of 5.62 (95% CI = 1.60 to 19.78)^26^ and 8.60 (95% CI = 3.86 to 19.13).^27^

The self-assessed questionnaire (SAQ) by Mimoun *et al* is a red flag for ectopic pregnancy, with an LR+ of 12.20 (95% CI = 3.03 to 49.81) for a score of >70. A score <25 on the SAQ can help rule out ectopic pregnancy (LR-0.19, 95% CI = 0.07 to 0.53).^28^ This questionnaire consists of questions concerning need for change of sanitary towels, type of discharge, duration of bleeding, and pain characteristics.

Other signs for diagnosing ectopic pregnancy, for example, estimated gestational age <70 days (LR-0.19, 95% CI = 0.05 to 0.75) and absence of foetal heart tones (LR-0.0, 95% CI = 0.00 to 3.55), met our criteria.^25,26^ Similarly, the combination of absence of foetal heart tones, no tissue at the cervical os, and abdominal tenderness had an LR- below 0.2 (LR-0.08, 95% CI = 0.01 to 0.58)^27^ (see Supplementary Appendix S2).

#### Clinical diagnosis

The clinical diagnosis was made by the attending physician (which could be an ED physician, surgeon, or not specified), without having access to results of blood tests or imaging tests. It was thus solely based on history taking and physical examination.

In one study, the clinical impression of the treating physician (not further specified) was a red flag for obstruction, diverticulitis, and appendicitis with an LR+ of 24.60 (95% CI = 11.43 to 52.95), 8.99 (95% CI = 6.17 to 13.10), and 10.62 (95% CI = 7.27 to 15.51), respectively.^21^ Other studies did not meet our prespecified LR criteria.

#### Other

Only 14 signs and symptoms, described above, had the capacity to either rule in or rule out target conditions causing acute abdominal pain. All other signs and symptoms listed in Supplementary Appendix S3, such as intense pain, persistent pain, left lower quadrant pain, history of fever, and speed bump sign, did not meet our predefined LR criteria.

## Discussion

### Summary

We summarised 16 studies on signs and symptoms of serious illness in adults presenting to ambulatory care settings with acute abdominal pain. Serious illnesses included were appendicitis, pelvic inflammatory disease, ectopic pregnancy, bowel obstruction, and diverticulosis. Of the 79 index test identified, only 14 signs and symptoms met our prespecified criteria of LR+ >5 or LR- <0.2, in at least one study. They include pain characteristics; systemic signs such as low systolic BP and shock index >0.85; gynaecological signs and symptoms such as cervical motion tenderness; SAQs; and the clinician’s overall assessment. For many tests that are routinely taught and used for assessing patients with abdominal pain, we either did not find any evidence (for example, absence of bowel movements), or found LRs that did not meet our predefined criteria (for example, history of fever, rebound tenderness, guarding, and right lower quadrant pain).

Importantly, the clinician’s overall assessment (clinical diagnosis), has by far the best rule-in capacity.^21,30,31^ This suggests that clinicians use a more integrated approach, incorporating multiple pieces of diagnostic information. Individual signs and symptoms are of limited accuracy when assessing acute abdominal pain,^36^ but when combined they may be more accurate. However, no studies evaluated multiple clinical features as a prediction rule.

Overall, we found more signs and symptoms that met our LR+ criterion than our LR- criterion, suggesting signs and symptoms are more useful as a red flag than for ruling out. This is particularly important in low incidence settings, where the likelihood of a serious illness is very low and it will be difficult to lower this even further. This means that clinicians should be actively looking for red flags that may increase the low likelihood of serious illness and therefore may change their management, but should not take the absence of such red flags as a sign to exclude a serious illness.

### Strengths and limitations

We only included prospective studies, applied specific inclusion and exclusion criteria, and performed a transparent and standardised quality assessment.^37^ However, heterogeneity was substantial on all important parameters of clinical diversity (variability in pre-selection of patients, definition of acute abdominal pain, interventions, reference standards, and outcomes), methodological diversity, variability in study design, and a moderate-to-high risk of bias.

In addition, we restricted the review to studies performed in an ambulatory care setting to maximise applicability, but no studies were performed in general practice, limiting transferability to general practice. Applicability can also be impeded by country-specific differences in healthcare systems.^24,25,28^

In this review we only focused on patients without pre-existing abdominal diseases, such as malignancies, inflammatory bowel diseases, or history of abdominal surgery. Therefore, our results cannot be transferred directly to this specific patient population.

Despite our extensive search strategy, we only found a limited number of studies, index tests, studies per index test, and target conditions. Therefore, pooling of data was not possible and results were reported descriptively.

### Comparison with existing literature

In earlier studies, gut feeling has also been shown to be a strong red flag for serious illness, for example, for serious infections in children^19^ and cancer in adults.^38^ Gut feeling has been defined as a 'sense of alarm' perceived by clinicians because they are concerned about possible adverse outcomes, even though specific indications are lacking. It’s been shown to be more accurate than clinical impression that the illness is serious, suggesting this could also be evaluated in adults with acute abdominal pain.^39^

### Implications for research and practice

Diagnosis of abdominal pain remains a challenge for ambulatory care clinicians, who deal with high patient volumes and make decisions amid uncertainty,^40^ resulting in missed, delayed, or wrong diagnoses.^41^ As mentioned above, individual signs and symptoms have little diagnostic ability and clinical diagnosis shows to be the strongest red flag for serious illness in patients with acute abdominal pain. Nonetheless, the vast majority of signs and symptoms, including classic textbook signs routinely taught to medical students, have little to no diagnostic value and scientific evidence. Students and clinicians should take this into account when assessing patients, especially the limited value of signs and symptoms in lowering the likelihood of serious illness. Considering this, more diagnostic tools for the ambulatory care setting are needed to support clinicians in their diagnostic work-up.

Considering the importance of acute abdominal pain in ambulatory care and the ubiquity of signs and symptoms in the evaluation of such patients, the low number of studies evaluating signs and symptoms is striking. This lack of evidence and heterogeneity prevented us from making firm conclusions. It emphasises the need for more research of better quality, in low prevalence settings, ideally using standardised methodology.

To conclude, we identified 14 signs and symptoms useful for diagnosing serious illness in patients presenting with acute abdominal pain. These include pain characteristics, systemic signs, gynaecological signs, and the clinician’s overall assessment. The latter being the best red flag, suggesting that clinicians combine clinical information in a more integrated approach. However, conclusions are based on only 16 studies, none of which were conducted in general practice. Better evidence in this setting is needed.
